# Effects of Shenkang Pills on Early-Stage Diabetic Nephropathy in db/db Mice *via* Inhibiting AURKB/RacGAP1/RhoA Signaling Pathway

**DOI:** 10.3389/fphar.2022.781806

**Published:** 2022-02-11

**Authors:** Fujing Wang, Jia’er Fan, Tingting Pei, Zhuo’en He, Jiaxing Zhang, Liliang Ju, Zhongxiao Han, Mingqing Wang, Wei Xiao

**Affiliations:** Department of Traditional Chinese Medicine, Southern Medical University, Guangzhou, China

**Keywords:** diabetic nephropathy, shenkang pills, LC/MS, transcriptome, AURKB, RacGAP1, RhoA

## Abstract

Diabetic nephropathy (DN) is the leading cause of end-stage renal disease, so there is an urgent need to suppress its development at early stage. Shenkang pills (SKP) are a hospital prescription selected and optimized from effective traditional Chinese medicinal formulas for clinical treatment of DN. In the present study, liquid chromatography-quadrupole-time of flight-mass spectrometry (LC-Q-TOF-MS) and total contents qualification were applied to generate a quality control standard of SKP. For verifying the therapeutic effects of SKP, db/db mice were administered intragastrically with SKP at a human-equivalent dose (1.82 g/kg) for 4 weeks. Moreover, the underlying mechanism of SKP were analyzed by the renal RNA sequencing and network pharmacology. LC-Q-TOF-MS identified 46 compounds in SKP. The total polysaccharide and organic acid content in SKP were 4.60 and 0.11 mg/ml, respectively, while the total flavonoid, saponin, and protein content were 0.25, 0.31, and 0.42 mg/ml, respectively. Treatment of SKP significantly reduced fasting blood glucose, improved renal function, and ameliorated glomerulosclerosis and focal foot processes effacement in db/db mice. In addition, SKP protected podocytes from injury by increasing nephrin and podocin expression. Furthermore, transcriptome analyses revealed that 430 and 288 genes were up and down-regulated in mice treated with SKP, relative to untreated controls. Gene ontology enrichment analysis revealed that the differentially expressed genes mainly involved in modulation of cell division and chromosome segregation. Weighted gene co-expression network analysis and network pharmacology analysis indicated that aurora kinase B (AURKB), Rac GTPase activating protein 1 (RacGAP1) and SHC binding, and spindle associated 1 (shcbp1) might be the core targets of SKP. This protein and Ras homolog family member A (RhoA) were found overexpression in db/db mice, but significantly decreased with SKP treatment. We conclude that SKP can effectively treat early-stage DN and improve renal podocyte dysfunction. The mechanism may involve down-regulation of the AURKB/RacGAP1/RhoA pathway.

## 1 Introduction

Diabetic nephropathy (DN) is a common chronic complication of diabetes mellitus (Gross et al., 2005). Increased urinary protein is a sign of early-stage of DN, which correlates with impaired glomerular filtration, and the renal function damage progression (Mogensen et al., 1983). Persistent proteinuria and a decrease in glomerular filtration rate gradually lead to end-stage renal disease. Thus, development of new drugs and identification of targetable signaling pathways mediating early-stage DN will be critical to control the morbidity and mortality associated with DN.

**GRAPHICAL ABSTRACT:**
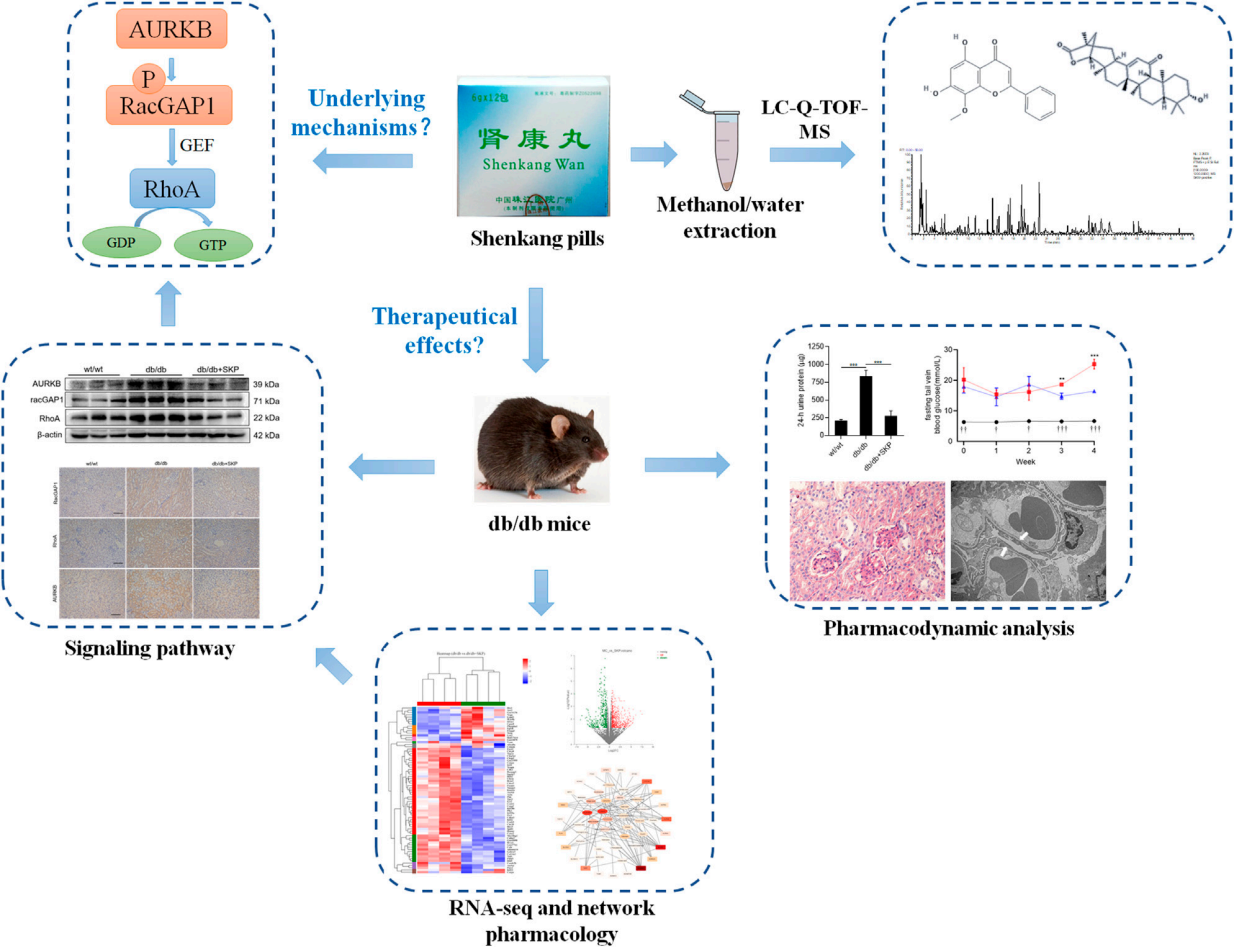
Study design.

Increased amounts of urinary protein are associated with functional and morphological alterations of podocytes. There is accumulating evidence that disorders of multiple signaling pathways such as Wnt/β-catenin (Dai et al., 2017), mammalian target of rapamycin (mTOR) (Xiong and Zhou, 2019), cytosolic extracellular signal-regulated kinase (ERK) (Zeng et al., 2019), and Ras homolog family member A (RhoA)/Rho kinase (Peng et al., 2019), occur during DN. RhoA is a member of the Rho family of small GTPases (Rho GTPases), which have important roles in actin cytoskeleton regulation (Matsuda et al., 2021). It is well-documented that excessive activation of RhoA and its downstream Rho-Kinase, induces glomerulosclerosis by causing podocytes dysfunction, renal epithelial-mesenchymal transition, and matrix upregulation in mesangial cells (Patel et al., 2005; Peng et al., 2008; Zhu et al., 2011). Moreover, RhoA is also a molecular switch that controls a series of signal transduction pathways, including ERK, and Wnt/β-catenin (Krepinsky, 2003; Kim et al., 2018). These signaling pathways are widely involved in DN (Zhang L. et al., 2019; Kim et al., 2017). However, the signaling pathways downstream of RhoA have been studied extensively, while upstream regulating pathways remained unexplored. Aurora kinase B (AURKB) is a highly conserved serine/threonine kinase that mainly functions to regulate chromosomal segregation during mitosis (Goldenson and Crispino, 2015). Overexpression of AURKB is observed in various malignancies and associated with poor prognosis (Bertran-Alamillo et al., 2019; Poulard et al., 2019; Nie et al., 2020). AURKB has been studied extensively in the context of oncology; however, its role in DN remains unknown.

Shenkang Pills (SKP) are a hospital prescription which is selected and optimized from effective traditional Chinese medicinal formulaes for clinical treatment of DN, according to Traditional Chinese Medicine (TCM) theory. SKP are comprised of eight individual Chinese herbs: *Astragalus membranaceus*, *Euryale ferox Salisb*, *Rosa laevigata Michx*, *Zea mays L*, *Whitemania pigra Whitman*, *Leonurus japonicus Houtt*, *periostracum cicadae*, and *Crataegus pinnatifida Bge*. Previous clinical study has shown that SKP can significantly improve the 24-h urine protein, fasting blood glucose, 2-h postprandial blood glucose, and glycosylated hemoglobin in patients with early DN (Ai-Cheng et al., 2009). As traditional Chinese medicines contain multiple components, determination of the main components active in treatment of DN is challenging. A liquid chromatography-quadrupole-time of flight-mass spectrometry (LC-Q-TOF-MS) system has provided a technological method to analyze the complex components of SKP. Although SKP has been used clinically for many years, the mechanism of it treating DN have not been clarified, which limits the further promotion and application of SKP.

In this study, An LC/MS analysis was applied and different total contents within SKP were detected for quality control of SKP. We next investigated the effects of SKP on db/db mice and the underlying mechanism in treating DN. RNA sequencing was conduct to explore differentially expressed genes (DEGs). To confirm the sequencing results, expression levels of AURKB, Rac GTPase activating protein 1 (RacGAP1), and RhoA were assessed to verify whether SKP could ameliorate DN by inhibition of the AURKB/RacGAP1/RhoA signaling pathway.

## 2 Materials and Methods

### 2.1 The Fingerprinting of Shenkang Pills by LC-Q-TOF-MS

SKP, obtained from Zhujiang Hospital (Guangzhou, China) (batch number: 181217), were powdered and passed through an 80 mesh (180 μm) sieve. Then, 0.5 g of the powder was accurately weighed and dissolved into 1 ml of a methanol: water solution (v/v, 3:1) for extraction. The suspension was sequentially vortexed (2 min), sonicated (40 Hz, 30 min), and centrifuged at 15,000 g for 20 min. The supernatant was then collected for HPLC-UV-Q Exactive analysis. Analyses were performed using an LC system (Thermo U3000, Thermo Fisher Scientific, MA, United States) coupled to a mass spectrometer system (Q Exactive, Thermo Fisher Scientific, MA, United States). For the LC/MS separation, the sample was analyzed in both the ESI-positive and -negative modes using a 3.0 × 100 mm, 2.7 μm column (Thermo), column temperature, 35°C. The mobile phase contained A = 2 mM ammonium formate +0.01% FA and B = acetonitrile + methanol (1:1). The flow rate was 0.4 ml/min, and 2 μl was injected into the system. The following gradient protocol was used: 0–1 min (5% B), 1–42 min (5% B), 42–45 min (45% B), 45–45.1 min (90% B), and 45.1–50 min (90% B). The total run time was set at 50 min. LC grade acetonitrile and methanol were acquired from Merck (Darmstadt, Germany). Other reagents and chemicals were analytical grade. The ion source was H-ESI and TOF data were collected between m/z 100 and 1,500 Da, in both the ESI- and -negative modes. Positive and negative ion capillary voltages were 3,500 and 2800 V, respectively. Vaporizer temperature was set at 350°C. Accurate mass and composition of the precursor and fragment ions were calculated using Compound Discoverer 3.1 software (Thermo).

### 2.2 Quantification of Total Polysaccharides, Organic acids, Flavonoids, Saponins and Proteins

#### 2.2.1 Quantification of Total Polysaccharides and Organic Acids

Total polysaccharides contents were determined using the colorimetric method described in the China Pharmacopeia. Total organic acid content within SKP was examined according to a previous method (Huang et al., 2007). The extracted polysaccharide supernatant was taken as a sample of total organic acids. Samples were collected and enriched using a rotary evaporator under reduced pressure at 60°C. An appropriate volume of anhydrous methanol was added to dissolve the sample and the volume adjusted to 25 ml with methanol. Absolute ethanol was added to a 0.5 ml extract and mixed to a final volume of 5 ml. Sodium lauryl sulfate (0.3%, 2.0 ml) and 1.0 ml 0.6% ferric chloride: 0.9% potassium ferricyanide (= 1:0.9) solution were added to the mixture and placed in the dark for 5 min. Acetic acid solution (1 mol/L) then was added to 25 ml, and the sample placed in the dark for 20 min. The absorbance of the mixture at 700 nm was examined by UV spectrophotometry. Each sample was determined for three times. The calibration curve was generated with caffeic acid.

#### 2.2.2 Quantification of Total Flavonoids

Total flavonoids were determined as a previously described study (Guijuan et al., 2004). Briefly, 2 g of the powder, as described above was extracted using 70% v/v aqueous ethanol in a 250 ml heat reflux reactor at 100°C for 2.5 h. The volume ratio of sample to solvent was 1:20. The extracted solution was collected and enriched using a rotary evaporator under reduced pressure at 60°C. Then, the concentrated sample was extracted 3 times with water-saturated ethyl acetate. Ethyl acetate was removed using a rotary evaporator and 30% v/v aqueous ethanol added to 25 ml. A 5% v/v sodium nitrite solution was added to 6 ml of extract and mixed for 6 min. After addition of 1 ml aluminum nitrate solution (5% v/v) and incubation for 6 min, 10 ml sodium hydroxide (1 mol/L) was added and diluted with 10 ml of water. The absorbance of the mixture at 500 nm was examined by UV spectrophotometry. Each sample was analyzed three times. A calibration curve was generated using rutin.

#### 2.3.3 Quantification of Total Saponins

Total organic acids content within SKP was examined according to a previous method (Guijuan et al., 2004). The aqueous layer was separated from ethyl acetate and extracted three times with water-saturated n-butanol. The n-butanol portion was concentrated by evaporation under reduced pressure and dissolved in absolute ethanol. Next, 0.75 ml vanillin (8%) was added to 0.5 ml extract and immediately cooled in an ice bath for 20 min. After addition of 7.5 ml sulfuric acid (72%), the mixture was incubated at 62°C for 20 min in a water bath. The absorbance of the mixture at 544 nm was examined by UV spectrophotometry. Each sample was analyzed three times. A calibration curve was generated using Astragaloside IV.

#### 2.3.4 Quantification of Total Protein

A previous method was referred to determine the total protein of SKP (Tao et al., 2018). In short, 2 g of the powder as described above was extracted by ultrasound (200 W, 40 kHz, and 40°C) in 75% ethanol (28:1, V/m). Then 5.0 ml of alkaline copper was added to the extraction and left standing at room temperature for 30 min. After added alkaline copper (1 mol/L), the mixture was placed at room temperature for 30 min. The absorbance at 500 nm was obtained from the UV spectrophotometer. The calibration curve was generated with bovine serum albumin.

### 2.3 Animals and Experimental Design

Seven-week-old male BKS-Lepr^em2Cd479^/Nju mice (db/db mice) and C57BLKS/JNju mice (wt/wt mice; controls) were purchased from GemPharmatech Co., Ltd. (Guangzhou, China) and the Experimental Animal Center of Southern Medical University, respectively. All mice were housed in a temperature-controlled room (22°C) under a 12 h light/12 h dark cycle, with free access to water and food. After 1 week of adaptive feeding, db/db mice were randomly divided into two groups: the db/db model group (*n* = 6), which was orally treated with saline water and the db/db + SKP group (*n* = 6), which was intragastrically administered with SKP at 1.82 g/kg for 4 weeks. The selection of SKP dosage was based on a recommended dose of SKP of 12 g/d for an adult human (60 kg) in clinical application, which equates to 0.2 g/kg; a human equivalent dose of 0.2 × 9.1 (the conversion coefficient) = a mouse dose of 1.82 g/kg (Nair and Jacob, 2016). Body weight and fasting blood glucose (GLU) were determined weekly during the experiments. All animal studies were approved by the Institutional Animal Care and Use Committee for Southern Medical University [usage license number SYXK (Yue) 2016-0167; certification number SCXK (Su) 2018-0008; SCXK (Yue) 2016-0041]. Animals were killed after 4 weeks of treatment, blood samples collected, and kidney tissues harvested.

### 2.4 Renal RNA Extraction, Library Preparation and Sequencing

Total RNA (*n* = 4 samples per group) was isolated from kidney tissues using Triquick Reagent (Trizol Substitute) (Solarbio, Beijing, and China), according to the manufacturer’s instructions. RNA quality and quantity were determined using a Kaiao K5500^®^ Spectrophotometer (Kaiao, Beijing, China) and an RNA Nano 6000 Assay Kit with the Bioanalyzer 2100 system (Agilent Technologies, CA, United States). Samples that met the criteria of OD260/280 = 1.8–2.2, OD260/230 ≥ 2.0, RIN ≥6.5, 28S:18S ≥ 1.0 were used to construct sequencing libraries.

Sequencing libraries were prepared using an NEBNext^®^ Ultra™ RNA Library Prep Kit for Illumina^®^ (#E7530L, NEB, United States). Raw sequencing reads from the Illumina platform were processed to obtain high-quality sequences (clean reads) by removing low-quality sequences and connector contamination. All subsequent analyses were based on clean reads.

Clean reads were separately aligned to the *Mus musculus* reference genome (GRCm38. p6, http://asia.ensembl.org/Mus_musculus/Info/Index) using HISAT2 software (http://ccb.jhu.edu/software/hisat2/index.shtml) (Kim et al., 2015). Mapped reads from each sample were assembled using StringTie (https://ccb.jhu.edu/software/stringtie/index.shtml) (Pertea et al., 2015). RSEM (http://deweylab.biostat.wisc.edu/rsem/) (Li and Dewey, 2011) was used to quantify gene abundance values. Differential expression analysis was performed using DEseq2 (Love et al., 2014) (*p-*value < 0.05); DEGs with fold-change (FC) ≥ 1.5 were considered to be significantly differentially expressed. In addition, Gene Ontology (GO) functional-enrichment analyses and GO annotation and classification of DEGs were performed using a Bonferroni-corrected *p*-value threshold of <0.05.

### 2.5 Gene Module Clustering Based on Weighted Gene Co-Expression Network Analysis

Eight sequencing samples and six extracted samples were sorted into three groups: wt/wt (*n* = 4), db/db (*n* = 4), and db/db + SKP (*n* = 4). After the transcriptome matrix was filtered to exclude transcripts with low expression levels and repeatedly merged, the weighted gene co-expression network analysis (WGCNA) method was used to construct a pharmacological network. First, co-expressed genes were clustered and modules identified (Supplementary Figure S1A). According to the clustering results, a total of 139 co-expressed gene modules were obtained and entered in the next step of analysis. To further optimize the speed of network construction, we used correlation analysis to merge co-expression models of the same type. Correlation analysis took the PC1 value after dimensionality reduction of the internal gene principal component analysis (PCA) of the module, and merged modules with correlation coefficient ≥0.8. A total of 79 modules were entered the next analysis phase (Supplementary Figure S1B). After dimensionality reduction by PCA, the PC1 principal component was taken, and Spearman correlation analysis conducted within each group. The results presented in Supplementary Figure S1D show that 12 of the 79 modules were positively correlated with the SKP group, while 1 module was negatively correlated (*p* < 0.01), with module 132 having the highest correlation with SKP (*ρ* > 0.99). Module 132 contained 88 genes and was entered into the next step of network construction.

### 2.6 Network Pharmacology Analysis

The compounds of SKP were listed in Supplementary Table S1, and the chemical structure were downloaded from pubchem (https://pubchem.ncbi.nlm.nih.gov/). The gene targets of these compounds were obtained from swisstargetprediction (http://www.swisstargetprediction.ch/). The target genes for diabetic kidney disease in this study were collected from Gene Cards (https://www.genecards.org/, version 5.6.0). Cytoscape software (v.3.9.0, https://cytoscape.org/) was used to visually analyze these data to construct component-target network. These common targets will lead to STRING (https://string-db.org/, version 11.5) predicting the associated protein–protein interaction (PPI).

### 2.7 Blood and Urine Analyses

GLU levels were measured weekly in blood from tail veins using a glucose meter (Sinocare, Changsha, China). Final GLU levels were measured in Nanfang Hospital (Guangzhou, China). Levels of serum creatine (Creatinine Assay Kit #C011-2-1), urea nitrogen (Blood Urea Nitrogen Assay Kit #C013-2), and glycated serum protein (GSP) (GSP Assay Kit #A037-2-1) were detected using assays from Jiancheng Bioengineering Institute (Nanjing, China). Mice were placed in metabolic cages for 24-h urine collection, and urine protein concentration detected using a Urine Protein Quantitative Kit #C035-2 (Jiancheng Bioengineering Institute, Nanjing, China).

### 2.8 Histological Analyses

For transmission electron microscopic (TEM) analyses, Mice kidney samples were fixed in ice-cold glutaraldehyde (2.5% in 1× phosphate buffer, pH 7.4) (17,003-92, Nacalai, Japan) at 4°C overnight. Then, samples were fixed in 1% osmium tetroxide for 2 h, dehydrated in ethanol, and embedded in epon resin. Samples were then sliced into 60-nm ultrathin sections and stained with uranyl acetate. Representative areas were analyzed using a Hitachi H-7500 (Hitachi, Tokyo, and Japan).

Kidneys were fixed in 4% phosphate-buffered formaldehyde, dehydrated in a gradient of ethanol, and then embedded in paraffin. Sections (4 μm) were cut consecutively, deparaffinized in xylene, rehydrated in graded concentrations of ethanol, and stained with hematoxylin and eosin (H&E) and periodic acid Schiff (PAS) for histological evaluation. A glomerular matrix index value was determined for each glomerulus using the following criteria: 0, normal; 1, <25% of the glomerulus; 2, 25–50% of the glomerulus; 3, 50–75% of the glomerulus; 4, 75–100% of the glomerulus. Twenty randomly chosen glomeruli were scored in each kidney section. Antibodies against AURKB (ab216341, abcam, Cambridge, United Kigdom, 1:200), RacGAP1 (13739-1-AP, Proteintech, Wuhan, China), and RhoA (10749-1-AP, Proteintech, Wuhan, and China) were used for immunohistochemical analysis (IHC).

For immunofluorescence, paraffin sections were deparaffinized, rehydrated, and antigen recovered in sodium citrate buffer by microwaving (10 min, 500 W). Then, specimens were permeated in cold methanol for 10 min, followed by washing three times in phosphate-buffered saline with Tween 20 (PBST), blocking with 5% goat serum in PBST for 1.5 h, and incubation with primary antibody against nephrin (1:200) overnight at 4°C. Sections were then washed thoroughly in PBST and incubated with DyLight 488-labeled goat anti-rabbit IgG (1:500) (Abbkine, Wuhan, China) in the dark for 1 h at room temperature. After washing with PBST, slides were observed under a fluorescence microscope (Nikon Eclipse 80i, Japan), and immunofluorescence intensity quantified using ImageJ software.

### 2.9 Real-Time Quantitative Polymerase Chain Reaction

cDNA was synthesized from 1 µg of total RNA using a PrimeScript RT reagent kit (RR047A, Takara, Tokyo, and Japan) and a thermal cycler. cDNA samples were quantified using SYBR Green (RR420A, Takara SYBR^®^ Premix Ex Taq^TM^), according to the manufacturer’s instructions. The specific primer sequences used were as follows: *Gadph*, Forward, GGT​TGT​CTC​CTG​CGA​CTT​CA, Reverse, TGG​TCC​AGG​GTT​TCT​TAC​TCC; *Aurkb*, Forward, CGG​TTC​AAC​AGC​CAG​TCC​ACA​G, Reverse, GCC​CAA​AGG​ACG​CCC​AAT​CTC; *Racgap1*, Forward, GAA​GTC​AGG​ACC​TTT​ACA​ACC​T, Reverse, CCC​AAA​TTG​TCT​GTG​TCA​GTT​C; *Shcbp1*, Forward, GTG​GGC​AGT​CTG​GCA​CAC​TAA​TG, Reverse, CAA​GCC​TTA​CGA​CCG​CCT​TCA​G RT-qPCR was conducted using a Roche LightCycler96 (Roche, Switzerland). Amplifications were carried out for 45 cycles (40 s at 95°C, 30 s at 60°C, and 60 s at 65°C). The experiment was repeated three times; average values are reported. Relative gene expression was calculated by the $2^{-\text{ΔΔ}C_{\text{q}}}$ method.

### 2.10 Protein Extraction and Western Blotting

Kidney tissue samples were thoroughly homogenized in RIPA lysis buffer (P0013B, Beyotime Biotech Co., Ltd., Shanghai, China) at low temperature. The protein concentrations in supernatants were measured using a BCA Protein Assay Kit (P0012S, Beyotime, Shanghai, and China). Protein aliquots (20 μg) were separated by 8–15% SDS-PAGE, transferred onto polyvinylidene difluoride membrane (Merck Millipore, United States), and blocked in 5% skim milk in Tris-buffered saline with Tween 20 (TBST). Then, blots were incubated overnight at 4°C in primary antibody solution containing anti -nephrin (ab216341), -AURKB (ab2254, Abcam, Cambridge, United Kigdom), podocin (20384-1-AP), -RacGAP1 (13739-1-AP), -RhoA (10749-1-AP), -Shcbp1 (12672-1-AP; 1:1,000) (all from Proteintech, Wuhan, China), or -GADPH (1:2,000, AF7021, Affinity, Jiangsu, China). Then, blots were incubated with horseradish peroxidase-conjugated goat anti-rabbit secondary antibodies (1:1,000, 7074S, Cell Signaling Technology, MA, United States) for 2 h at 4°C. Protein bands were visualized using an ECL chemiluminescence reagent kit (WBKLS0100, Millipore, MA, United States). The average optical density of bands was quantified using ImageJ (National Institutes of Health, Bethesda, United States).

### 2.11 Statistical Analyses

Experimental data are expressed as the mean ± SEM. One-way ANOVA and subsequent Tukey’s post-test were used to determine the significance of differences in multiple comparisons. Histological scores were analyzed using the Kruskal–Wallis test, followed by the Mann-Whitney U test for multiple comparisons. Statistical analyses were conducted using SPSS software version 23.0 (IBM, New York, NY, and United States). *p* values <0.05 were considered statistically significant.

## 3 Results

### 3.1 Quality Control of Shenkang Pills by LC-Q-TOF-MS

**FIGURE 1:**
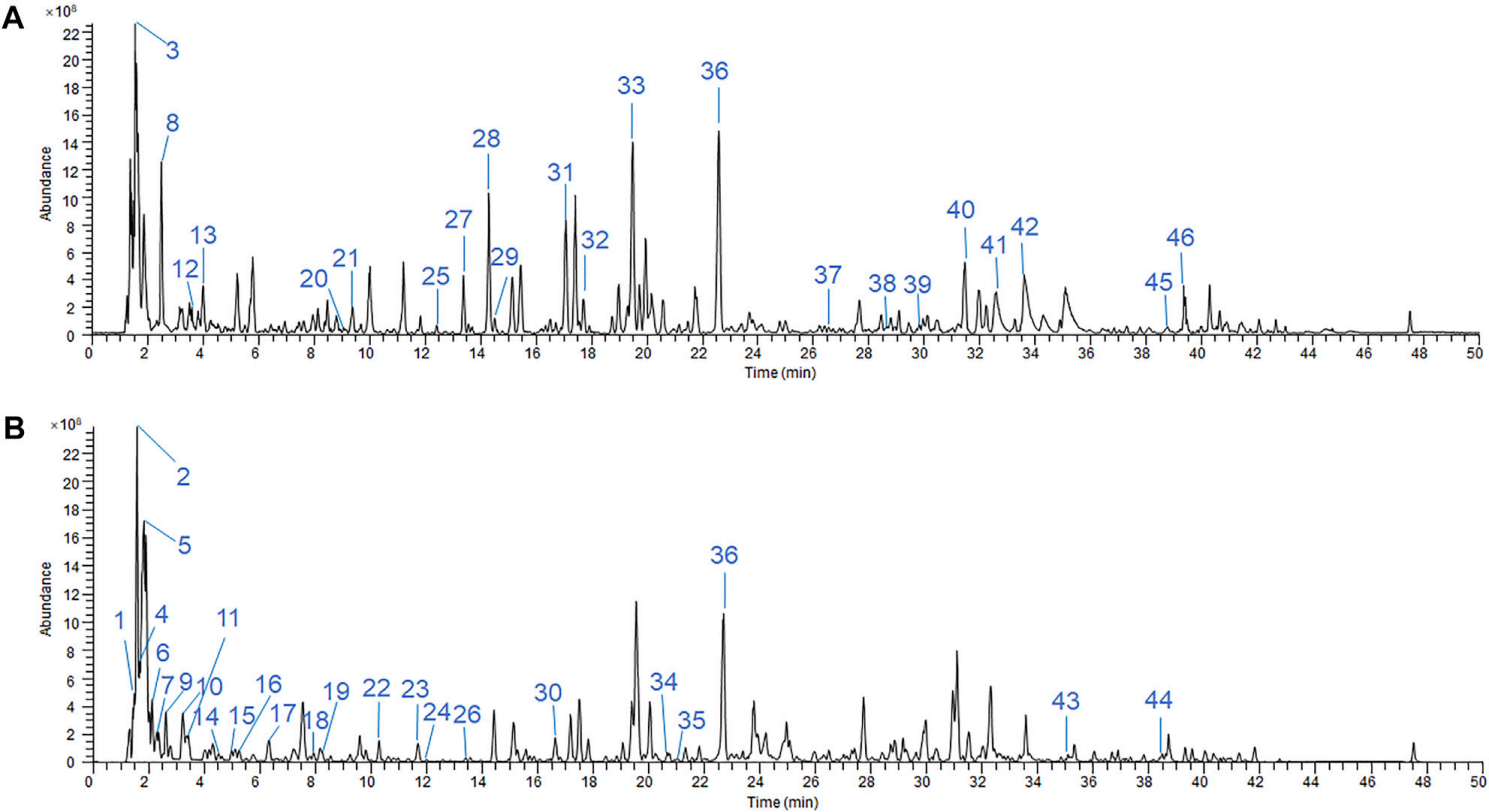
LC-Q-TOF-MS/MS chromatographic fingerprinting of SKP in ESI-positive mode **(A)** and ESI-negative mode **(B)**. LC condition: 3.0 × 100 mm, 2.7 μm column, run temperature at 35°C. Flow rate at 0.4 ml/min. The mobile phases were A = 2 mM ammonium formate +0.01% fomic acid and B = acetonitrile + methanol (1:1). MS condition: The TOF data had been collected between m/z 100 and 1,500 Da in both the ESI-positive and -negative modes.

The chromatographic quality control by LC-Q-TOF-MS is performed to obtain the fingerprinting of SKP. Base peaks in positive and negative ion mode from analysis of SKP are presented in Figures 1A,B. By comparing retention times and fragment patterns using compound discoverer software, 46 SKP compounds were identified. Retention time, parent ion, compound name, formula, error, and structure class data are presented in Supplementary Table S1. Besides formononetin, which was detected in both positive and negative ion modes, 21 compounds were detected in positive ion mode and 24 in negative ion mode. The 46 compounds identified in SKP included 14 organic acids, 9 flavonoids, 5 saponins, 4 purine derivatives, 2 alkaloids, and other classified compounds. Regarding the overall qualities of SKP, LC-Q-TOF-MS was used to analyze the alcohol-extracted fraction, while proteins, polysaccharides, and other components that cannot be detected in alcohol extracts were extracted and their concentrations determined using different methods. To understand differences between batches of SKP, three samples with different batch numbers were tested in triplicate. As shown in Table 1, the contents of total polysaccharides and organic acids in SKP were 4.60 and 0.11 mg/ml, respectively, while the contents of total flavonoids, saponins, and protein were 0.25, 0.31, and 0.42 mg/ml, respectively.

**TABLE 1:** Total contents of polysaccharides, organic acids, flavonoids, saponins, and protein within Shenkang pills.

Substance	Content (mg/ml)			Mean
Batch	180324	180528	181217	
Polysaccharides	5.38 ± 0.004	3.96 ± 0.008	4.46 ± 0.003	4.60 ± 0.003
Organic acids	0.10 ± 0.008	0.13 ± 0.016	0.10 ± 0.002	0.11 ± 0.020
Flavonoids	0.22 ± 0.001	0.19 ± 0.001	0.33 ± 0.004	0.25 ± 0.070
Saponins	0.42 ± 0.004	0.24 ± 0.003	0.26 ± 0.002	0.31 ± 0.095
Protein	0.40 ± 0.008	0.47 ± 0.001	0.40 ± 0.001	0.42 ± 0.043

### 3.2 SKP Reduced GLU and Improved Renal Function in db/db Mice

**FIGURE 2:**
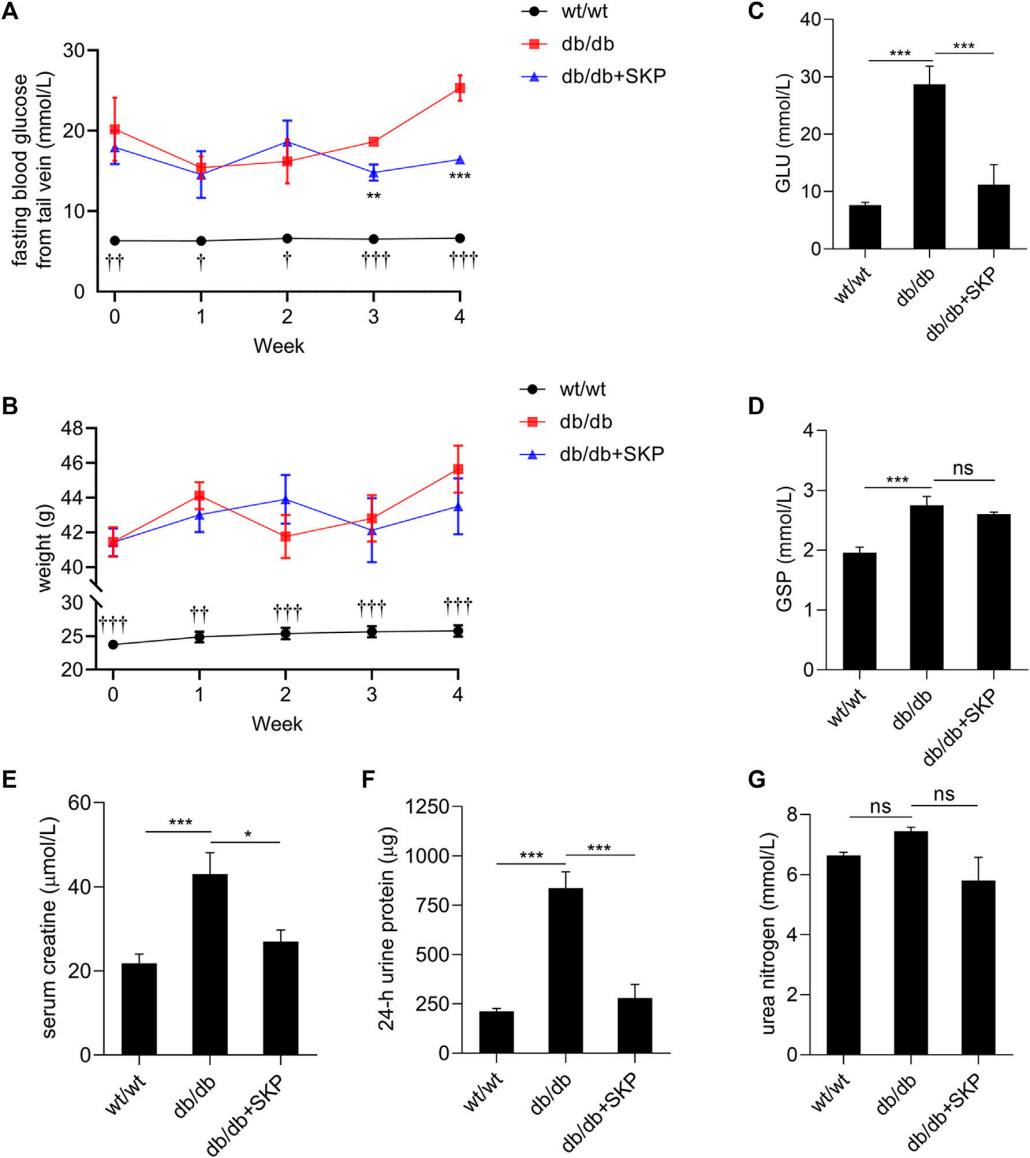
SKP reduced GLU and 24-h urine protein in db/db mice. **(A,B)** Sequential changes in body weight and GLU concentrations in the three groups of mice over the 4-week period. **(C,D)** GLU concentrations and GSP levels after 4 weeks of administration of SKP. **(E–G)** Serum creatine, 24-h urine protein and urea nitrogen. All values are shown as mean ± SEM, with levels of significance determined by ANOVA and a subsequent Tukey test. ^†^ *p* < 0.05 wt/wt group vs. db/db group; ^††^ *p* < 0.01 wt/wt group vs. db/db group; ^†††^ *p* < 0.001 wt/wt group vs. db/db group; ^**^ *p* < 0.01 db/db + SKP group vs. db/db group; ^***^ *p* < 0.001 db/db + SKP group vs. db/db group; ^*^ *p* < 0.05 and ^***^ *p* < 0.001 vs. the indicated groups. ns, no significance.

To examine the effects of SKP in db/db mice, GLU and body weight were recorded weekly during SKP administration (Figures 2A,B). As expected, GLU levels remained <10 mmol/L in the wt/wt group, and were three times higher in the db/db group than the wt/wt group (20.2 ± 9.65 vs 6.33 ± 0.33 mmol/L) at 8 weeks old, demonstrating that 8-week-old db/db mice have begun to develop diabetes. Before SKP administration, there was no difference in GLU between the db/db and db/db + SKP groups (*p* > 0.05); however, the GLU level in the db/db + SKP group began to decrease in the third week (*p* < 0.01), and fell markedly after 4 weeks of SKP treatment (*p* < 0.001) (Figure 2A **)**. Similarly, serum glucose levels were consistent with tail vein blood glucose in the fourth week (Figure 2C). To better understand the average blood glucose concentration in mice in the first to 3^rd^ weeks before they were euthanized, GSP levels were assessed and found to be higher in the db/db group than the wt/wt group (Figure 2D). After treatment with SKP, GSP levels decreased, although the difference was not significant (*p* = 0.15), indicating that mean blood glucose levels were increased in the db/db group and that SKP treatment induced long, slow development of hypoglycemia.

Simultaneously, db/db mouse weight was almost twice that of wt/wt mice (23.74 ± 1.62 vs 41.45 ± 0.33 g) at 8 weeks old and were always heavier over the 4-week period. However, treatment with SKP did not affect body weight (Figure 2B). To determine whether renal function changed in 12-weeks db/db mice, we next investigated serum creatine, urea nitrogen, and 24-h urine protein levels. The results showed that serum creatine levels in db/db group mice were twice higher than those in the wt/wt group (*p* < 0.001). Further, treatment with SKP remarkably decreased the level of serum creatine in the db/db + SKP group (*p* < 0.05) (Figure 2E), while 24-h urine protein also increased significantly in the db/db group (*p* < 0.001), and was significantly decreased in response to SKP treatment (*p* < 0.001) (Figure 2F). Nevertheless, there were no significant differences in urea nitrogen levels among the three groups (db/db vs db/db + SKP group, *p* = 0.051) (Figure 2G). These results indicate that 12-weeks db/db mice had developed diabetic renal injury, but were still in an early-stage of DN with just abnormally elevated urinary protein excretion. SKP treatment reduced GLU and protected the kidneys from injury in db/db mice.

### 3.3 SKP Ameliorated Renal Pathological Injuries in db/db Mice

**FIGURE 3:**
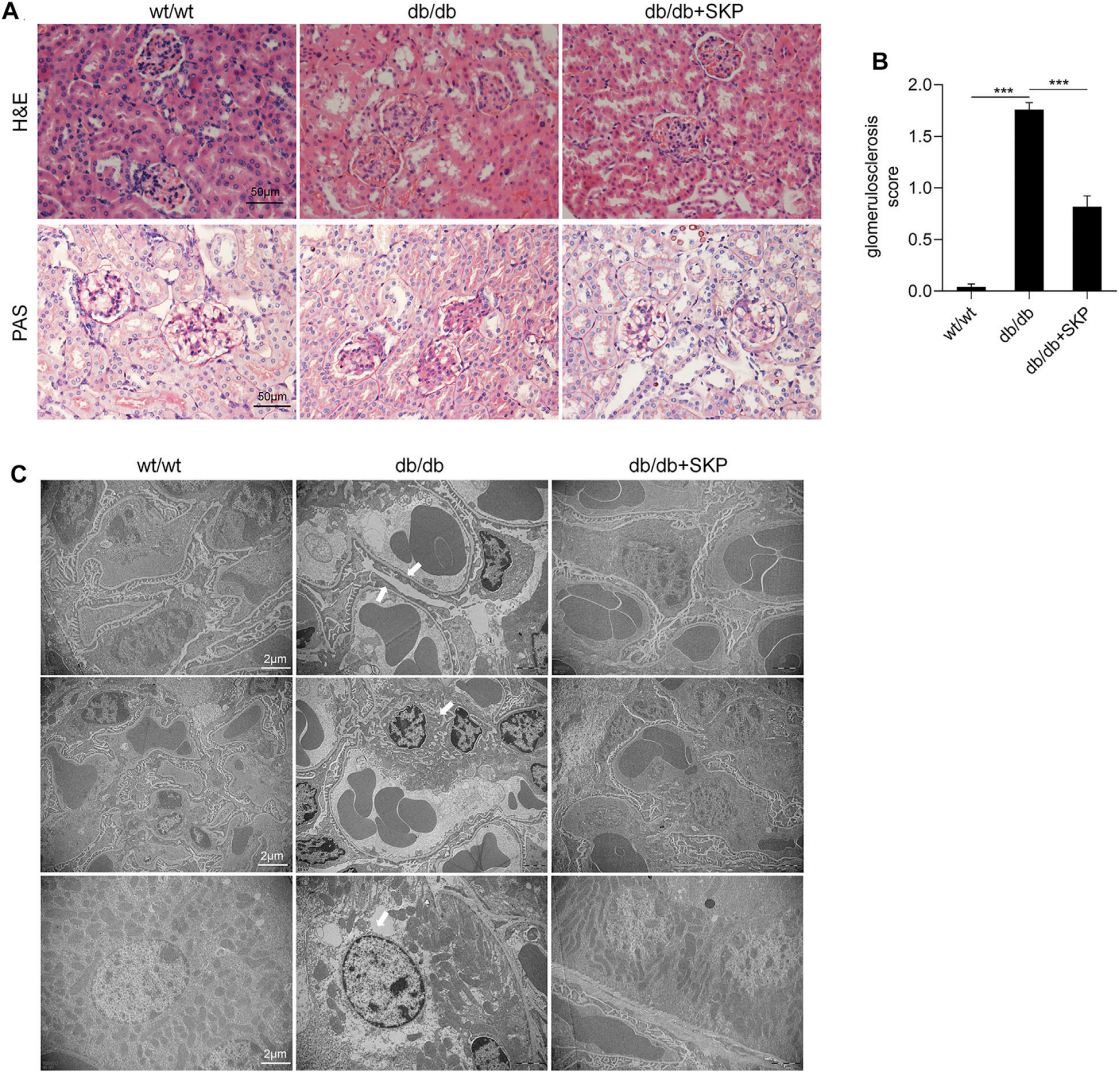
SKP ameliorated renal pathological injury in db/db mice. **(A)** Representative pictures of H&E staining and PAS staining. Original magnification ×400. **(B)** Quantitative evaluation of glomerulosclerosis by PAS staining. **(C)** Representative transmission electron microscopic analyses of glomeruli and tubular. Original magnification ×10,000. All values are shown as mean ± SEM, with levels of significance determined by ANOVA and a subsequent Tukey test. ^***^ *p* < 0.001 vs. the indicated groups.

As shown by H&E staining, glomeruli and tubules exhibited a normal structure in the wt/wt group, while distinct glomerulosclerosis, tubular dilatation, vacuolar degeneration of renal tubular epithelial cells, and were noted in the db/db group. By contrast, these renal pathological injuries were ameliorated in db/db mice treated with SKP (Figure 3A). To further assess glomerulosclerosis, PAS staining was used to visualize areas of glomerulosclerosis more clearly. Glomerular hypertrophy and increased glycogen were prominent in db/db mice, while changes alleviated in db/db mice treated with SKP (Figure 3A). Moreover, the glomerulosclerosis score increased from 0.04 ± 0.03 in the wt/wt group to 1.76 ± 0.07 in the db/db group (*p* < 0.001), while treatment with SKP significantly decreased this to 0.81 ± 0.10 (*p* < 0.001) (Figure 3B).

TEM was used to observed the ultrastructure of pathology of the kidneys. Figure 3C showed representative photomicrographs of kidney sections of the three groups. TEM of glomeruli in the db/db group showed obvious focal foot process effacement which was remarkably alleviated following treatment with SKP. Proteinuria is associated with podocyte injury, which is a key determinant of glomerulosclerosis (Kopp et al., 2020), where effacement of the podocyte foot processes causes podocyte detachment and a decrease in their number (D'Agati et al., 2011). Interestingly, the glomerular basement membrane (GBM) did not thicken in any of the three groups. Besides, mild hyperplasia of the mesangial matrix and occasional renal tubular epithelial cell necrosis were found in the kidney sections. Collectively, these findings indicate that SKP have no obvious difference in mesangial hyperplasia and basement membrane thickening, but more prominent in improving glomerulosclerosis, and podocyte injury.

### 3.4 SKP Up-Regulated Nephrin and Podocin Expression

**FIGURE 4:**
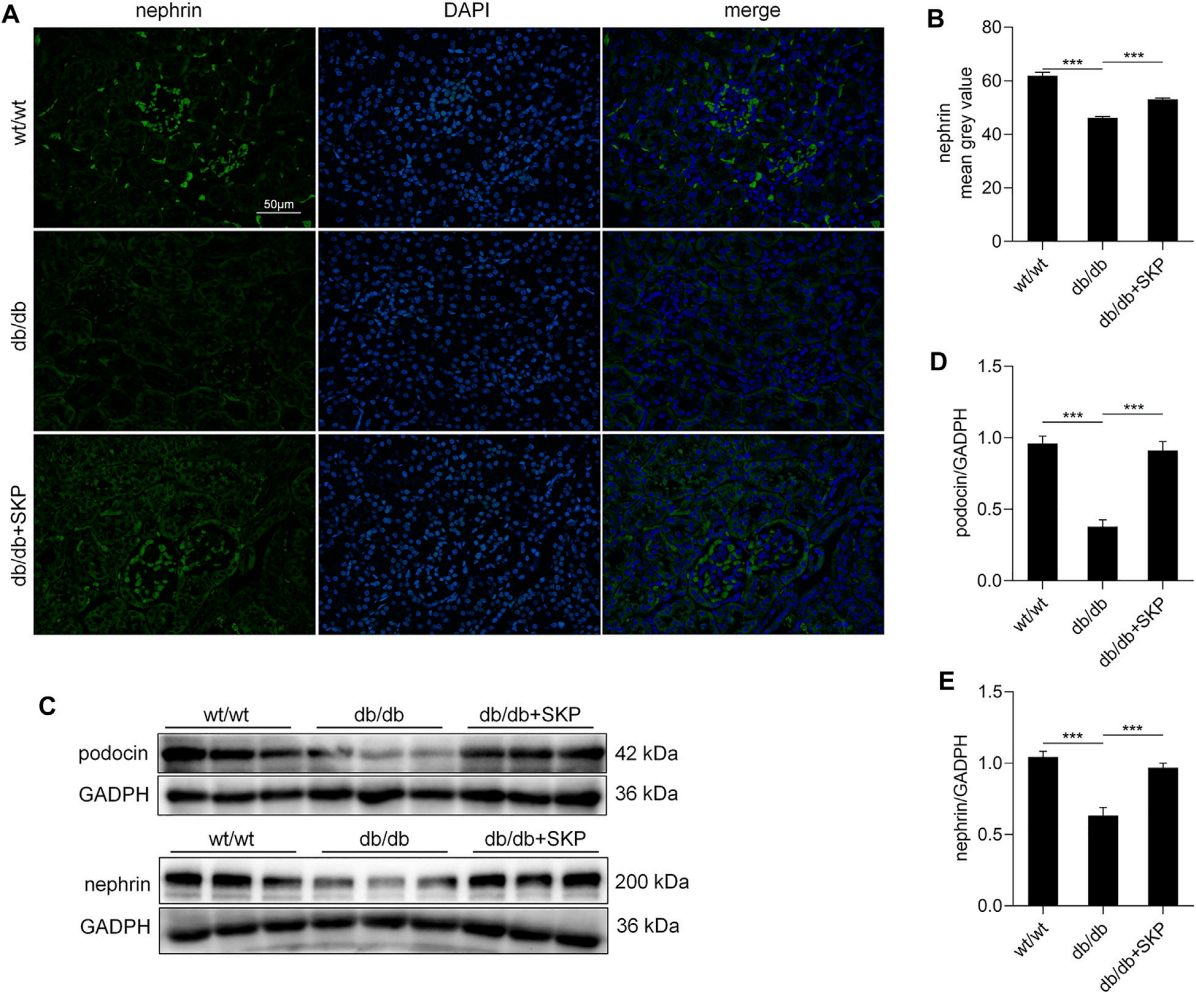
SKP up-regulated the expression of nephrin and podocin. **(A)** Nephrin expression in glomeruli by immunofluorescence staining. Original magnification ×400. **(B)** fluorescence intensity of nephrin measured by image J. **(C)** Western blots for podocin and nephrin expression. **(D,E)** Quantitative ratios of podocin and nephrin to GADPH. All values are shown as mean ± SEM, with levels of significance determined by ANOVA and a subsequent Tukey test. ^***^ *p* < 0.001 vs. the indicated groups.

To further evaluate podocyte injury, nephrin and podocin protein were examined by immunofluorescence staining and western blotting. Podocin (also referred to as NPHS2) and nephrin are key functional components of the slit diaphragm of podocytes, which is the structurally molecular filter in renal glomerular capillaries (Schwarz et al., 2001; Wartiovaara et al., 2004). Their absence results in loss of the slit diaphragm, effacement of the podocyte foot processes, and severe proteinuria (Pavenstädt et al., 2003; Kopp et al., 2020). Restoring the expression of these two proteins contributes to recovery of podocyte function and reduction of proteinuria (Vaughan et al., 2005). The immunofluorescence staining showed that fewer nephrin-positive areas were observed in the db/db group than in the wt/wt group, while treatment with SKP led to partial recovery of the immunofluorescence intensity (Figure 4A). Quantitative analysis of the immunofluorescent intensity of nephrin staining demonstrated a decrease in db/db mice (*p* < 0.001), that was partially restored by SKP treatment (*p* < 0.001) (Figure 4B). Likewise, the trend in levels of podocin was similar to that of nephrin (Supplementary Figures S2A, B). Further, the results of western blotting for nephrin and podocin proteins were consistent with those generated by immunofluorescence staining (Figures 4C–E). Overall, these results suggested that SKP may play a role in the protection of podocytes.

### 3.5 Identification of Differentially Expressed Genes by RNA Sequencing

**FIGURE 5:**
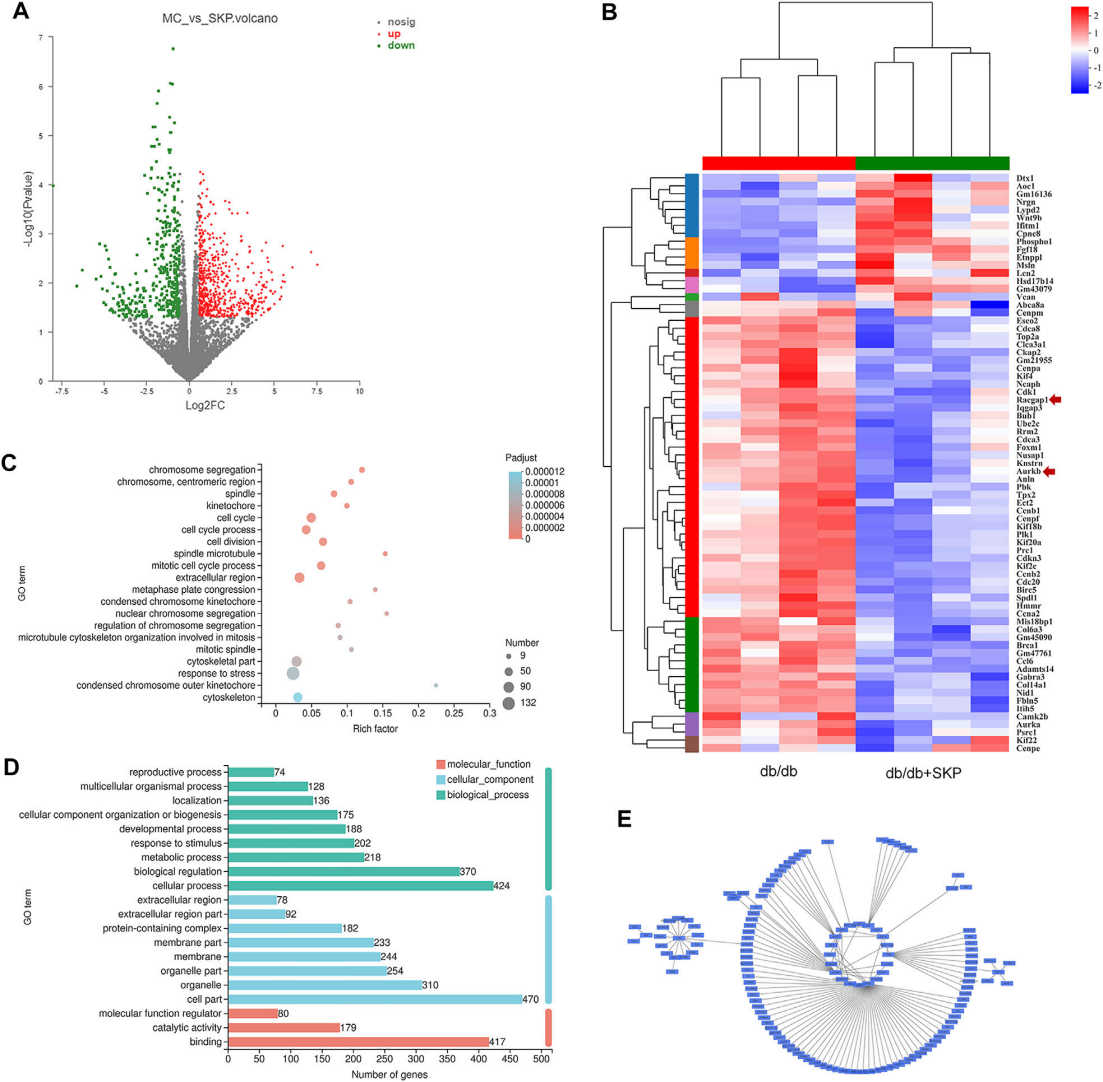
Differentially expressed genes (DEGs) by RNA sequence. **(A)** Volcano plots of DEGs for db/db + SKP group compared with db/db group (FC = fold change, FC > ± 1.5, *p*-value < 0.05). Grey dots = no change; red dots = upregulated; green dots = downregulated. **(B)** Heatmap for comparison between db/db and db/db + SKP group, and the expressions of *racgap1* and *Aurkb* were as indicated by an arrow. Changed the parameter of significance level from *p*-value < 0.05 to *P*-adjust < 0.05, and FC from > ± 1.5 to > ± 2.0. **(C)** Gene Ontology (GO) enrichment analysis of DEGs. (FC > ± 1.5, *p-value* < 0.05) **(D)** GO functional classification (FC > ± 1.5, *p*-value < 0.05). **(E)** A network of modules (module 132) related to the mechanism of SKP constructed based on WGCNA.

To investigate the mechanism underlying the renoprotective effect of SKP against DN, we performed transcriptome profiling of kidney samples from db/db mice treated with and without SKP. The results revealed significant differences in the expression of 718 genes between the two groups treated with or without SKP (FC > ± 1.5, *p* < 0.05), with 430 and 288 genes up- and down-regulated, respectively (Figure 5A). GO enrichment analysis showed that the DEGs were highly enriched in functions including cell division, chromosome segregation, spindle microtubule, and kinetochore, among others (Figure 5B). Annotation and classification of DEGs using the GO database also revealed that they were mainly involved in the modulation of cell parts (*n* = 470), cellular processes (*n* = 424), binding (*n* = 417), and biological regulation (*n* = 370) (Figure 5C). To illustrate DEGs with high FC values, we adjusted the threshold from ±1.5 to ±2.0 and generated a heat map (Figure 5D), which identified 16 upregulated and 57 downregulated DEGs (FC > ± 2.0, *p* < 0.05).

**FIGURE 6:**
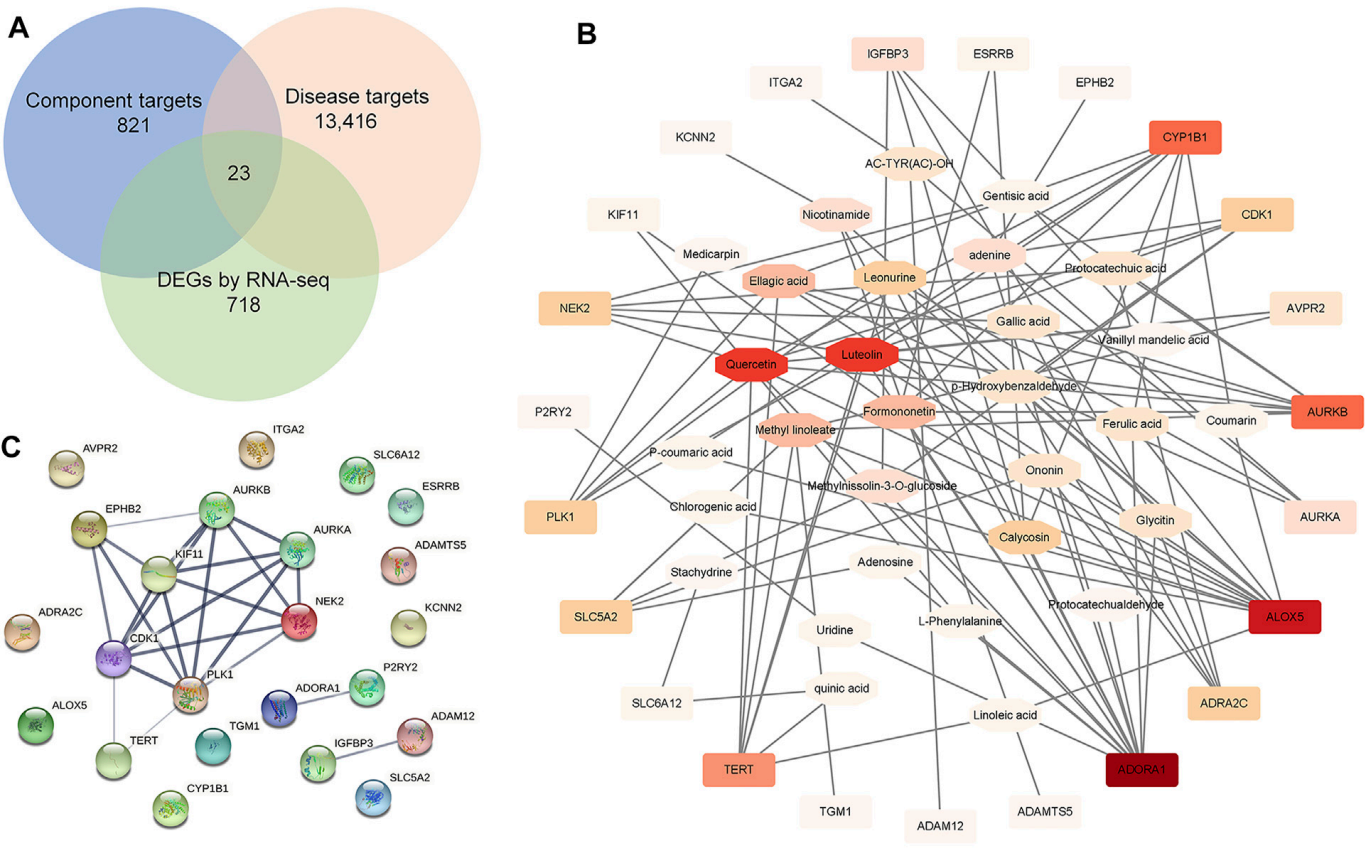
Network pharmacology analysis. **(A)** A Venn diagram showed the intersection of the component targets of SKP, DN disease targets, and DEGs by RNA-seq for common targets analysis. **(B)** A component-target network constructed by Cytoscape software. **(C)** An associated protein–protein interaction (PPI) diagram predicted by String website.

Next, WGCNA and network pharmacology analysis were performed to further determine the core target of SKP. After gene module clustering based on WGCNA (Supplementary Figures S1A, B), a total of 88 genes in a SKP related module (module 132) were used to construct a network, according to the correlation coefficients, and the network was transformed to scale-free by pruning low correlations (Figure 5E). The centrality, degree and closeness of the network were analyzed, and the nodes with the maximum likelihood of three parameters selected. The results suggested that four genes: *Racgap1, Shcbp1*, *Psrc1*, and *Dscaml1* were screened out, and may be core targets of SKP. To better understand the relationship between the components of SKP and these DEGs, a component-target network was constructed by network pharmacology analysis. We took the intersection of the component targets of SKP, DN disease targets, and DEGs for common targets analysis (Figure 6A). Among the 23 common target genes, *Adora1* (15), *Alox5* (14), *Cyp1b1* (8), and *Aurkb* (8) were with the highest degree value. There were eight components in SKP, namely ellagic acid, formononetin, gallic acid, gentisic acid, luteolin, methyl linoleate, protocatechuic acid, and quercetin, all targeting *Aurkb* (Figure 6B). The PPI diagram revealed that *cdk1* (14)*, plk1* (14)*, Aurkb* (12) interacted with many other proteins, and may be the core targets of SKP (Figure 6C; Supplementary Figure S1C).

### 3.6 SKP Inhibited the AURKB/RacGAP1/RhoA Signaling Pathway

Based on the results of the GO enrichment analysis, heatmap, WGCNA, and network pharmacology, *Aurkb*, *Racgap1*, and *Shcbp1* were chose for verification of acting targets of SKP. AURKB, a novel target in cancer, mediates phosphorylation of shcbp1, while RacGAP1 regulates cell division (Minoshima et al., 2003; Asano et al., 2013). The RT-qPCR analysis showed that mRNA levels of these genes were higher in the db/db than the wt/wt group (*p* < 0.001). Further, treatment with SKP reversed the increases of those genes (*p* < 0.001) (Supplementary Figures S2C–E). Consistent with the literature, the results described above suggest that SKP down-regulates AURKB and its downstream genes.

**FIGURE 7:**
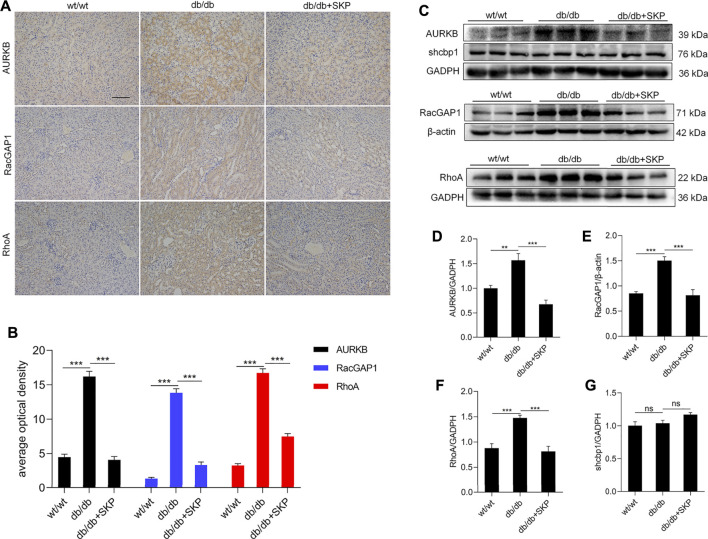
SKP inhibited AURKB/RacGAP1/RhoA signaling pathway. **(A)** Representative pictures of immunohistochemical assays for AURKB, RacGAP1 and RhoA. Original magnification ×200. **(B)** Semiquantitative measurements of tubulointerstitial areas: average optical density analysis (AOD) of AURKB, RacGAP1 and RhoA. **(C)** Western blots for AURKB, shcbp1, RacGAP1 and RhoA. **(D)** Quantitative ratios of AURKB to GADPH. **(E)** Quantitative ratios of RacGAP1 to β-actin. **(F)** Quantitative ratios of RhoA to GADPH. **(G)** Quantitative ratios of shcbp1 to GADPH. All values are shown as mean ± SEM, with levels of significance determined by ANOVA and a subsequent Tukey test. ^**^ *p* < 0.01 and ^***^ *p* < 0.001 vs. the indicated groups. ns, no significance.

Moreover, AURKB colocalizes with RacGAP1 and RhoA, suppresses the interaction of Shcbp1 with RacGAP1, and promotes RhoA activation (Zhang G.Y. et al., 2019). To further investigate these proteins in DN, we used IHC to determine the expression localization of AURKB, RacGAP1, and RhoA. IHC of AURKB suggested that there was some staining for AURKB in renal sections from the wt/wt group; however, AURKB levels were significantly higher, particularly in tubulointerstitium of db/db mice (*p* < 0.001), and administration of SKP clearly decreased this expression (*p* < 0.001) (Figures 7A,B). Hence, AURKB levels showed a consistent trend with IHC (Figures 7C,D). IHC of RacGAP1 and RhoA also showed that there was weak staining in the wt/wt group, indicating low expression levels. By contrast, the levels of RacGAP1 and RhoA were mainly increased in renal tubulointerstitial sections from db/db mice (*p* < 0.001). Likewise, areas that stained positive for RacGAP1 and RhoA were reduced in response to SKP administration (*p* < 0.001) (Figures 7A,B). Similarly, RacGAP1 and RhoA protein levels were elevated in the db/db group and reduced in response to SKP, indicating that SKP exerted renoprotective effects by down-regulation of RacGAP1 and RhoA (Figures 7C,E,and F). By contrast, the expression of Shcbp1 showed no apparent change in any of the three groups (Figures 7C,G).

## 4 Discussion

DN is described as “Xiao ke”, “Shen xiao” or “Niao zhuo” in TCM. The symptoms of DN include frequent micturition, with profuse urine, urine turbidity, edema, sore waist and knees, fatigue, dizziness, tinnitus, pale tongue, white tongue coating, and thin and weak pulse. DN pathogenesis is essentially empty and out solid. Specifically, empty of spleen and kidney is substantial reason, while dampness and blood stasis are resulting phenomena. Therefore, the therapeutic principle for DN in TCM is to invigorate the spleen and kidney, promoting blood circulation, and removing dampness and turbidity. SKP is a prescription for clinical treatment of DN from Zhujiang Hospital (Guangzhou, China), that obtained a national invention patent (Patent No: ZL 200610036515.2). In the theory of TCM, SKP exerts its treatment effects on DN by strengthening the spleen and tonifying the kidney, activating blood circulation, and eliminating dampness.

A total of 46 peaks in the positive and negative modes have been identified corresponding compounds from the fingerprints obtained by LC-MS. Formononetin, calycosin, ononin, chlorogenic acid, ferulic acid, medicarpin, methylnissolin-3-O-glucoside, nicotinamide, coumarin, astragaloside IV, astragaloside II, and calcifediol are components of *A. membranaceus*, a Qi-tonifying Chinese herbal medicine. Formononetin has been proven to control hypoglycemia, reduce insulin resistance, and act as an antioxidant in diabetic animals (Oza and Kulkarni, 2019; Zhuang et al., 2020). Calycosin can protect from renal injury caused by inflammatory cytokines, oxidative stress, and fibrotic processes in high fat diet-fed/streptozocin injected rats (Elsherbiny et al., 2020). Chlorogenic acid has been shown to exert hypoglycemic, hypolipidemic, antioxidant, and anti-inflammatory effects (Yan et al., 2020). Ferulic acid protects hyperglycemia-induced kidney injury by antioxidant, anti-inflammatory, and anti-apoptotic (Chowdhury et al., 2019). Coumarin derivative attenuated oxidative stress and fibrosis induced by high glucose in mesangial cells (Yao et al., 2020). Astragaloside IV can also ameliorate the renal injury in DN (Wang et al., 2020). Nevertheless, ononin, medicarpin and methylnissolin-3-O-glucoside have rarely been studied in the context of kidney disease. Protocatechuic acid is an extract from *Z. mays* and *R. laevigata* that exerts anti-oxidation and antiglycation effects, and reduces extracellular matrix accumulation *in vivo* and *vitro* (Harini and Pugalendi, 2010; Lin et al., 2011; Ma et al., 2018). P-coumaric acid, from *Z. mays*, can reduce serum glucose, improve renal function, and restore oxidant/antioxidant balance in DN rats (Zabad et al., 2019). Stachydrine, a compound from *L. japonicus*, inhibits the deleterious effects of high-glucose levels on endothelial cells (Servillo et al., 2013). To summarize, SKP contains a variety of constituents that can ameliorate diabetes and improve kidney function.

C57BLKS/J db/db mice are widely used as a model of progressive DN, because of their obvious albuminuria and mesangial matrix expansion (Sharma et al., 2003). In db/db mice, blood glucose levels begin to be elevated at 4 weeks of age and develop hyperglycemia at 8 weeks old (Lee and Bressler, 1981), which is consistent with our results. Renal dysfunction caused by glomerulosclerosis is the main reason for DN progression and db/db mice kidneys have enlarged glomeruli, with increased mesangial matrix reported at 12-weeks old (Sharma et al., 2003); however, GBM thickening is primarily noted in diabetic mice older than 12 months (Sharma et al., 2003). Similarly, in our study, clear areas of glomerulosclerosis were detected in the kidneys of db/db mice. Nevertheless, no obvious GBM thickening was observed in any mice, also consistent with previous findings, as discussed above. Podocyte injury is a critical mediator of glomerulosclerosis and closely correlated with nephrotic proteinuria (Deegens et al., 2008). Moreover, the slit diaphragm formed by the foot processes of podocytes is a core component of the glomerular filter (Pavenstädt et al., 2003). Podocyte injury leads to effacement of the podocyte foot processes and loss the markers of slit diaphragm: nephrin and podocin (Lenoir et al., 2015). Our results indicated that SKP treatment protected podocyte from injury by up-regulation of nephrin and podocin. Among the 46 components of SKP, there are eight components that have been reported to protect podocytes and increase the expression of nephrin or podocin: Astragaloside IV restored the expression of podocin and nephrin and attenuated podocyte apoptosis in cultured mouse podocytes (Chen et al., 2008). Astragaloside II ameliorated podocyte injury with nephrin restoration in streptozotocin (STZ)-induced diabetic rats (Su et al., 2021). Ferulic acid up-regulated the expressions of nephrin and podocin proteins in STZ-induced DN rats (Qi et al., 2020). Quercetin was observed improvement of the kidney ultrastructure, and tissue mRNA of podocin in a pristane-induced mouse model of lupus nephritis (Dos et al., 2018). Ellagic acid prevented the advanced glycation end products-mediated loss of expression nephrin and podocin in diabetic rats (Raghu et al., 2016). Oleanolic acid combined with Protosappanin-A increased protein levels of nephrin, podocin, and CD2AP in sC5b-9-induced podocyte apoptosis (Zheng et al., 2019). Calcitriol treatment resulted in restoration of nephrin signalling in the STZ-diabetic animal model (Trohatou et al., 2017). Leonurine suppressed adriamycin-induced reduction in the expression of nephrin and podocin (Liu et al., 2018).

Transcriptomic and network pharmacology analysis of DEGs indicated that they were mainly involved in modulation of cell division and chromosome segregation. Therefore, mitotic related genes were validated by RT-qPCR and western blotting, including: *Aurkb*, *Shcbp1*, and *Racgap1*. Previous study has demonstrated that AURKB phosphorylation of shcbp1 suppresses the interaction of shcbp1 with RacGAP1 (Zhang G. Y. et al., 2019). In the present study, the expression of mRNA and protein of AURKB and RacGAP1 were significantly increased in the db/db group. However, mRNA expression of *shcbp1* increased but without difference in protein expression, possibly due to the phosphorylation of it. Aurora kinase B (AURKB) is a member of serine/threonine kinases regulating mitosis, especially the process of chromosomal segregation (Goldenson and Crispino, 2015). Overexpression of AURKB has been clarified related to a range of cancers including clear cell renal cell carcinoma (Wan et al., 2019), gastric cancer (Nie et al., 2020), leukemia (Poulard et al., 2019), and lung cancer (Bertran-Alamillo et al., 2019). AURKB promotes survival of cancer cells by cell cycle progression with phosphorylating a series of downstream substrates, including RacGAP1(Tang A. et al., 2017). A study of gene coexpression network analysis identified that AURKB might be valuable biomarkers of gestational diabetes mellitus (Zhao and Li, 2019). Our results of the network pharmacology analysis also revealed that AURKB was targeted by eight components in SKP and interacted with many other proteins. However, the role of AURKB in DN is unknown due to few studies.

Podocytes are terminally differentiated cells and cannot repair themselves through cell division (Wiggins, 2007). Nephrin and podocin are key functional components of the slit diaphragm and markers of differentiated podocytes (Schwarz et al., 2001; Wartiovaara et al., 2004). Loss of mitotic activity is accompanied by phenotypic conversion, with expression of slit membrane-associated proteins, including nephrin, podocin, and several other specific proteins (Pavenstädt et al., 2003). In response to high glucose in DN, differentiated podocytes re-enter the cell cycle and undergo mitotic catastrophe, which is a major cause of podocyte loss (Hara et al., 2019). The up-regulation of these mitotic genes (*Aurkb*, *RacGAP1* and *shcbp1*) suggested that podocytes in the db/db group mice might have entered an abnormal mitotic condition. Similar to our results, an *in vitro* experiment found that cultured podocytes induced by high glucose showed enhanced expression of AURKB and other mitotic markers (Tang H. et al., 2017). Podocyte function is maintained by a well-organized actin cytoskeleton, which is disrupted into disorganized short filaments during foot process effacement (Faul et al., 2007). Mitogenic stimuli or DNA damage force podocytes to complete mitosis; however, mature podocytes cannot replicate and maintain their actin cytoskeleton simultaneously, leading to incomplete formation of mitotic spindles, aberrant chromosome segregation, and/or podocyte detachment during mitosis (Liapis et al., 2013). AURKB phosphorylation of Caspase-2 is a key step in mitotic catastrophe to trigger apoptosis of mitotically defective cells (Lim et al., 2021). Nevertheless, the relationship between AURKB and the podocyte actin skeleton is not completely understood.

Interestingly, RacGAP1 phosphorylation by AURKB, a GTPase-activating protein, stimulates its activity towards RhoA, thus promoting cytokinesis (Zhang G. Y. et al., 2019). In this study, overexpression of RhoA was found in the kidneys of db/db mice. RhoA is a member of the Rho GTPases, which are master regulators of the actin cytoskeleton (Matsuda et al., 2021). Among the 22 Rho GTPase family members, RhoA promotes formation of contractile actin- and myosin-containing stress fibers (Blaine and Dylewski, 2020). Excessive activation of RhoA in podocytes leads to albuminuria and focal segmental glomerulosclerosis in humans (Zhu et al., 2011). RhoA and its immediate downstream target, Rho kinase (ROCK) widely involved in progression in DN (Kim et al., 2017; Rao et al., 2017). Astragaloside IV, calycosin, chlorogenic acid and ferulic acid are bioactive compounds from *Astragalus membranaceus*, all reported to inhibit RhoA excessive activation or its downstream ROCK signaling pathway in various cell and animal models (Jiang et al., 2015; Peng et al., 2018; Xie et al., 2020; Liao et al., 2021). Gallic acid, a component from *Crataegus pinnatifida Bge*, has been also found suppression of RhoA expression in vascular smooth muscle cell proliferation and migration (Chung et al., 2020). In addition, two components from *Whitemania pigra Whitman* (adenosine and vitamin D3), one blunted RhoA activation in pulmonary endothelium (Harrington et al., 2004), the other attenuated high glucose-induced activation of RhoA/ROCK pathway in HK-2 cells (Zhang et al., 2017).

## 5 Conclusion

In summary, the present study provides 46 major compounds and qualification of different contents for quality control of SKP. Treatment with SKP decreased GLU and improved renal function in db/db mice. At the pathological level, SKP ameliorated glomerulosclerosis and effacement of the podocyte foot processes by increasing the expression of nephrin and podocin. Furthermore, SKP treatment inhibited expression of AURKB, RacGAP1, and RhoA. Overall, we demonstrate for the first time that SKP ameliorates early-stage DN *via* the AURKB/RacGAP1/RhoA signaling pathway. Further experiments are needed to prove and further explore the role of these three proteins in DN.

## Data Availability

The original contributions presented in the study are publicly available. This data can be found here: BioProject, PRJNA766249.
