# Supplementary material for: Effects of Shenkang Pills on Early-Stage Diabetic Nephropathy in db/db Mice via Inhibiting AURKB/RacGAP1/RhoA Signaling Pathway
Source: Front Pharmacol. 2022 Feb 11;13:781806. doi: 10.3389/fphar.2022.781806 (PMC8873791; doi:10.3389/fphar.2022.781806)
Supplement: Supplementary file 1 [file DataSheet1.docx]

Supplementary Material

## Supplementary Tables

**Table S1** Identification of chemical compounds within SKP by LC-Q-TOF-MS.

| No. | Rt (min) | Aduct | Extraction mass (Da) | Error (ppm) | Formula | Identification | | MS^2^ ions | Source |
| --- | --- | --- | --- | --- | --- | --- | --- | --- | --- |
| 1 | 1.46 | - | 182.07897 | -0.36 | C_6_H_14_O_6_ | Mannitol | | (-) 181.07178 [M-H]^-^, 163.06107, 131.03505, 119.03487, 101.02430, 89.02435, 73.02943, 71.01378, 57.03459 | (Yingping, 2004) |
| 2 | 1.56 | - | 192.06350 | 0.57 | C_7_H_12_O_6_ | Quinic acid | | (-) 191.05621 [M-H]^-^, 173.04558, 127.04008, 111.00883, 93.03455, 87.00874, 85.02957, 71.01390, 59.01386 | (Qijiayu et al., 2021) |
| 3 | 1.58 | + | 143.09461 | -0.10 | C_7_H_13_NO_2_ | Stachydrine | | (+) 144.10175 [M+H]^+^, 84.08070, 58.06539 | (Huizhen et al., 2021) |
| 4 | 1.66 | - | 134.02145 | 0.48 | C_4_H_6_O_5_ | Malic acid | | (-) 133.01416 [M-H]^-^, 115.00360, 89.02434, 72.99300, 71.01377, 59.01383 | (Qijiayu et al., 2021) |
| 5 | 1.82 | - | 192.02716 | 0.81 | C_6_H_8_O_7_ | Citric acid | | (-) 191.05583 [M-H]^-^, 129.01926, 111.00876, 87.00877, 85.02955, 67.01895, 57.03456 | (Qijiayu et al., 2021) |
| 6 | 2.11 | - | 118.02658 | -0.24 | C_4_H_6_O_4_ | Succinic acid | | (-) 117.01933 [M-H]^-^, 99.00870, 96.32854, 91.06969, 73.02944, 67.84531, 55.01869 | (Qijiayu et al., 2021) |
| 7 | 2.28 | - | 244.06955 | 0.05 | C_9_H_12_N_2_O_6_ | Uridine | | (-) 243.06197 [M-H]^-^, 200.05667, 152.03545, 140.03517, 122.02458, 110.02470, 82.02979, 66.03484 | (Guoqiang et al., 2018) |
| 8 | 2.51 | + | 122.04793 | -0.68 | C_6_H_6_N_2_O | Nicotinamide | | (+) 123.05502 [M+H]^+^, 106.02866, 96.04413, 80.04933, 78.03383 | (Hui et al., 2020) |
| 9 | 2.60 | - | 135.05447 | -0.17 | C_5_H_5_N_5_ | Adenine | | (-) 134.04715 [M-H]^-^, 107.03629, 92.02520, 65.01458 | (Guoqiang et al., 2018) |
| 10 | 3.21 | - | 206.07918 | 0.69 | C_8_H_14_O_6_ | D-(-)-Tartaric Acid Diethyl Ester | | (-) 205.07179 [M-H]^-^, 143.07130, 129.05569, 115.07640, 99.08150, 81.07085, 72.99303 | (Hao, 2021) |
| 11 | 3.34 | - | 170.02147 | -0.31 | C_7_H_6_O_5_ | Gallic acid | | (-) 169.01418 [M-H]^-^, 125.02432, 97.02948, 81.03465, 69.01897 | (Hao, 2021) |
| 12 | 3.58 | + | 126.03161 | -0.70 | C6 H6 O3 | 5-Hydroxymethylfurfural | | (+) 127.03880 [M+H]^+^, 109.02835, 81.03343, 53.03894 | (Hao, 2021) |
| 13 | 3.99 | + | 267.09654 | -0.78 | C_10_H_13_N_5_O_4_ | Adenosine | | (+) 268.10339 [M+H]^+^, 136.06161, 119.03505, 57.03357, 55.01808 | (Guoqiang et al., 2018) |
| 14 | 4.51 | - | 154.02664 | 0.2 | C_7_H_6_O_4_ | Gentisic acid | | (-) 153.01935 [M-H]^-^, 125.02437, 123.04528, 109.02948, 81.03455 | (Qijiayu et al., 2021) |
| 15 | 4.79 | - | 154.02664 | 0.21 | C_7_H_6_O_4_ | Protocatechuic acid | | (-) 153.01938 [M-H]^-^, 140.85695, 132.66383, 109.02948, 91.01888, 81.03461, 74.13300, 65.03961, 53.77675 | (Qijiayu et al., 2021) |
| 16 | 5.24 | - | 265.09511 | 0.33 | C_13_H_15_NO_5_ | AC-TYR(AC)-OH | | (-) 264.08777 [M-H]^-^, 220.09746, 202.08755, 194.08235, 178.08736, 119.05036, 96.04558, 82.02989 | (Qijiayu et al., 2021) |
| 17 | 6.31 | - | 138.03160 | -0.68 | C_7_H_6_O_3_ | Protocatechualdehyde | (-) 137.02440 [M-H]^-^, 136.01651, 109.02962, 108.02181 | | (Lu et al., 2011) |
| 18 | 7.92 | - | 152.04729 | -0.38 | C_8_H_8_O_3_ | Vanillin | (-) 151.03999 [M-H]^-^, 136.01601, 124.01662, 111.00864, 109.02943, 107.05083 | | (Xiao-Rui et al., 2018) |
| 19 | 8.22 | - | 122.03671 | -0.56 | C_7_H_6_O_2_ | p-Hydroxybenzaldehyde | (-) 121.02942 [M-H]^-^, 108.02161, 95.01390, 93.03449, 92.02672, 91.01884 | | (Huizhen et al., 2021) |
| 20 | 9.06 | + | 354.095 | -0.23 | C_16_H_18_O_9_ | Chlorogenic acid | (+) 355.08817, 163.03876, 145.02832, 135.04390, 117.03333, 79.05432 | | (Hui et al., 2020) |
| 21 | 9.38 | + | 165.07897 | -0.05 | C_9_H_11_NO_2_ | L-Phenylalanine | (+) 166.08609 [M+H]^+^, 148.07594, 136.07558, 118.06502, 87.04403, 80.04941, 69.03366 | | (Peili et al., 2017) |
| 22 | 10.28 | - | 290.07921 | 0.61 | C_15_H_14_O_6_ | Epicatechin | (-) 289.07190 [M-H]^-^, 245.08183, 221.08197, 205.05063, 203.07170, 161.06059, 151.04010, 137.02400, 123.04505, 109.02946, 97.02928, 83.01381, 57.03453 | | (Yinglan et al., 2009) |
| 23 | 11.68 | - | 198.05286 | 0.19 | C_9_H_10_O_5_ | Vanillyl mandelic acid | (-) 197.04572 [M-H]^-^, 182.02211, 166.99875, 153.05582, 138.03232, 121.02956, 106.00608, 95.01401, 89.00328 | | (Hao, 2021) |
| 24 | 11.98 | - | 164.04740 | 0.60 | C_9_H_8_O_3_ | P-coumaric acid | (-) 163.04010 [M-H]^-^, 117.03444, 119.05016, 93.05502 | | (Xiao-Rui et al., 2018) |
| 25 | 12.41 | + | 146.0367 | -0.55 | C_9_H_6_O_2_ | Coumarin | (+) 147.04401 [M+H]^+^, 119.04907, 91.05415, 65.03871 | | (Jiao and Cong, 2018) |
| 26 | 13.34 | - | 194.05800 | 0.45 | C_10_H_10_O_4_ | Ferulic acid | (-) 193.05043 [M-H]^-^, 178.02715, 149.06081, 139.03989, 137.02440, 134.03740, 133.02957, 106.04258, 71.01383 | | (Hui et al., 2020) |
| 27 | 13.38 | + | 311.14784 | -0.89 | C_14_H_21_N_3_O_5_ | Leonurine | (+) 312.15488 [M+H]^+^, 181.04926, 132.11287, 114.10245, 97.07590, 72.08076 | | (Huizhen et al., 2021) |
| 28 | 14.29 | + | 446.12130 | 0.01 | C_22_H_22_O_10_ | Glycitin | (+) 447.30688 [M+H]^+^, 393.99710, 368.14249, 285.07541, 270.05197, 253.04921, 225.05449, 137.02328 | | (Shizhong, 2018) |
| 29 | 14.54 | + | 302.04262 | -0.11 | C_15_H_10_O_7_ | Quercetin | (+) 303.04980 [M+H]^+^, 257.04449, 241.19040, 229.04929, 201.05466, 165.01779, 153.01816, 137.02336, 69.06993 | | (Qian et al., 2018) |
| 30 | 16.62 | - | 302.00649 | 0.75 | C_14_H_6_O_8_ | Ellagic acid | (-) 300.99927 [M-H]^-^, 299.99149, 283.99643, 257.00961, 245.00887, 229.01442, 201.01939, 185.02454, 173.02449, 157.02928, 145.02962 | | (Qian et al., 2018) |
| 31 | 17.06 | + | 430.12617 | -0.49 | C_22_H_22_O_9_ | Ononin | (+) 269.08032 [M+H]^+^, 254.05760, 213.09077, 154.02628, 107.04928 | | (Yun-Feng et al., 2021) |
| 32 | 17.71 | + | 462.15242 | -0.38 | C_23_H_26_O_10_ | Methylnissolin-3-O-glucoside | (+) 480.18628 [M+NH_4_]^+^, 301.10641, 269.08029, 191.06998, 167.07007, 152.04666, 134.03604, 123.04385 | | (Li et al., 2020) |
| 33 | 19.47 | + | 284.06816/284.06846 | -1.09/-0.03 | C_16_H_12_O_5_ | Calycosin | (+) 285.07523 [M+H]^+^, 270.05170, 253.04910, 225.05440, 213.05440, 197.05937, 137.02316, 134.03606 | | (Yun-Feng et al., 2021) |
| 34 | 20.60 | - | 270.08928 | 0.25 | C_16_H_14_O_4_ | Medicarpin | (-) 269.04572 [M-H]^-^, 253.05083, 225.05624, 213.05611, 153.01945, 133.02950, 135.00876, 108.02168, 91.01898 | | (Bo et al., 2016) |
| 35 | 20.94 | - | 286.04796 | 0.76 | C_15_H_10_O_6_ | Luteolin | (-) 285.04068 [M-H]^-^, 217.05093, 175.04013, 151.00375, 149.02432, 133.02950, 107.01402, 65.00330 | | (Peili et al., 2017) |
| 36 | 22.58/22.67 | +/- | 268.07331/268.07369 | -0.91/0.47 | C_16_H_12_O_4_ | Formononetin | (+) 269.08057 [M+H]^+^, 254.05701, 253.04927, 237.05429, 226.06241, 213.09094, 197.05957, 137.02335, 118.04123, 107.04925 (-) 267.06638 [M-H]^-^, 252.04295, 251.03517, 224.04823, 223.04028, 195.04518, 135.00868, 132.02170, 91.01894 | | (Yun-Feng et al., 2021) |
| 37 | 26.55 | + | 244.10987 | -0.30 | C_15_H_16_O_3_ | Osthole | (+) 245.11639 [M+H]^+^, 189.05440, 159.04388, 131.04900, 103.05415 | | (Jing-Jing et al., 2018) |
| 38 | 28.59 | + | 784.46033 | 0.73 | C_41_H_68_O_14_ | Astragaloside IV | (+) 785.46741 [M+H]^+^, 437.34299, 419.32864, 185.13242, 143.10663, 125.09602, 71.04915 | | (Hui et al., 2020) |
| 39 | 29.80 | + | 826.47079 | -0.82 | C_43_H_70_O_15_ | Astragaloside II | (+) 827.47830 [M+H]^+^, 455.34824, 437.34097, 419.33121, 395.07358, 175.05991, 157.04936, 115.03883, 97.02825 | | (Li et al., 2020) |
| 40 | 31.45/31.98 | + | 294.25578 | -0.34 | C_19_H_34_O_2_ | Methyl linoleate | (+) 295.22668 [M+H]^+^, 277.21600, 179.14281, 151.11159, 105.06985, 93.06980, 81.03344, 67.05437, 55.05453 | | (Qijiayu et al., 2021) |
| 41 | 32.56 | + | 519.33264 | 0.29 | C_26_H_50_NO_7_P | 1-Linoleoyl-sn-glycero-3-phosphocholine | (+) 520.33911 [M+H]^+^, 184.07310, 124.99971, 104.10685, 86.09634, 60.08100 | | (Qijiayu et al., 2021) |
| 42 | 33.25 | + | 323.28227 | -0.49 | C_20_H_37_NO_2_ | Linoleoyl ethanolamide | (+) 324.28940 [M+H]^+^, 306.27911, 133.10106, 109.10109, 95.08540, 83.08556, 67.05432, 62.06023 | | (Qijiayu et al., 2021) |
| 43 | 35.06 | - | 280.24035 | 0.43 | C_18_H_32_O_2_ | Linoleic acid | (-) 279.23297 [M-H]^-^, 261.22595, 230.58789, 117.05947, 84.39149, 59.01382 | | (Qijiayu et al., 2021) |
| 44 | 36.01 | - | 456.36088 | 1.17 | C_30_H_48_O_3_ | Oleanolic acid | (-) 455.35385 [M-H]^-^, 407.33411, 296.90137, 252.27000, 147.00703, 124.61312, 92.09042, 65.05673 | | (Huizhen et al., 2021; Qijiayu et al., 2021) |
| 45 | 38.76 | + | 384.33901 | -0.54 | C_27_H_44_O | Vitamin D3 | (+) 385.30862 [M+H]^+^, 177.09070, 137.05943, 109.10118, 95.08536, 81.06982, 67.05437 | | (Shizhong, 2018) |
| 46 | 39.38 | + | 400.33385 | -0.70 | C_27_H_44_O_2_ | Calcifediol | (+) 401.34113 [M+H]^+^, 383.33017, 365.31918, 175.11168, 157.10097, 109.10114, 95.08537, 81.06980 | | (Jiao and Cong, 2018) |

Rt: retention time.

**References**

Bo, W., Wei, Z., Xiao-Hua, L., Xiao-Ya, L., Ya-Li, C., and Shi-Lan, F. (2016). Rapid Separation and Determination of 5 Kinds Flavonoids in *Astragali Radix* Based on Ultra-performance

Convergence Chromatography. Chinese J. Anal. Chem. 44(05), 731-739

Guoqiang, L., Yunyi, L., Tao, L., Yaolan, L., Guocai, W., Chunhua, W., and Zheng, L. (2018). Chemical constituents from Whitmania pigra. Jinan University*.* 35(09), 703-705

Hao, J. (2021). Content determination of ten organic acids in Crataegus pinnatifida before and after processing by HPLC. Tianjin Journal of Traditional Chinese Medicine*.* 38(07), 935-940

Hui, J., Shenglong, G., Yuting, Z., and Chang, F. (2020). Research progress on the chemical constituents and pharmacological effects of *Astragalus membranaceus*. Journal of Anhui Traditional Chinese Medical College*.* 39(05), 93-96

Huizhen, Y., Ying, L., and Xizhen, G. (2021). Research Progress on Chemical Components and Pharmacological Effects of *Lagopsis Supina*，*Leonurus japonicus* and *Prunella vulgaris*. Journal of Beijing Union University*.* 35(02), 85-92

Jiao, W., and Cong, W. (2018). Research progress on the chemical constituents and pharmacological effects of Astragalus. Journal of Xinxiang Medical College*.* 35(09), 755-760

Jing-Jing, Q., Qi-Nan, W., Min, X., Qian, W., Yi-Ming, X., and Da-Wei, W. (2018). Research progress on chemical components and pharmacological effects of *Leonurus japonicas*. Chinese Traditional and Herbal Drugs*.* 49(23), 5691-5704

Li, Z., Da-Wei, Q., Fan-Shu, B., Kai-Di, H., Sheng, G., Hui, Y., Zhen, O., Jian-Jun, Z., Jian-Qiang, Y., and Jin-Ao, D. (2020). Study on quality evaluation of *Astragali Radix* based on UPLC-MS. Chinese Journal of Pharmaceutical Analysis*.* 40(04), 722-732

Lu, Y., Guoyu, L., and Jinhui, W. (2011). Research status of the chemical constituents and pharmacological effects of *periostracum cicadae*. Journal of Nongken Medicine*.* 33(02), 184-186

Peili, Z., Yan, Z., Jinhai, H., and Weiming, W. (2017). General Situation of Research on Effective Chemical Constituents and Pharmacological Actions of *Zea mays L*. Heilongjiang Journal of Traditional Chinese Medicine*.* 46(01), 74-75

Qian, C., Na, L., Yulin, Z., He-Nan, L., and Guixia, H. (2018). Simultaneous determination of 7 polyphenols in *Rosa laevigata Michx* by HPLC. Journal of Chinese Medicinal Materials*.* 41(02), 394-396

Qijiayu, Z., Peiyuan, Z., Jing, S., Chongbo, Z., Yijun, S., and Jianhua, W. (2021). Research progresson chemical constituents and pharmacological action of hawthorn. Northwest Pharmaceutical Journal*.* 36(03), 521-523

Shizhong, X. (2018). Study of the changes of chemical composition of leech after processing. Shaanxi Journal of Traditional Chinese Medicine*.* 39(07), 980-982

Xiao-Rui, F., Rao-Rao, L., Li-Mei, L., Duan-Fang, L., and Chun, L. (2018). Research Progress in Medicinal Parts of *Rosa laevigata Michx*. Chinese Pharmaceutical Journal*.* 53(16), 1333-1341

Yinglan, Z., Cai, L., Hongjun, H., Li, D., and Bin, H. (2009). Content Determination of Epicatechin in Crataegolic from Different Producing Area. China Pharmaceuticals*.* 18(12), 34-35

Yingping, W. (2004). Chemical ComPonents and Their Antioxidant Activities Studies on Maize Silk (stigma maydis). Jilin Agricultural University.

Yun-Feng, Z., Yang, L., Wei-Ping, D., Chen-Guang, Z., Jie, S., Cun-Yu, L., and Gouping, P. (2021). Determination of isoflavonoids and glycosides in *Astragali Radix* by QAMS method. Chinese Traditional and Herbal Drugs*.* 52(10), 3104-3111

## Supplementary Figures


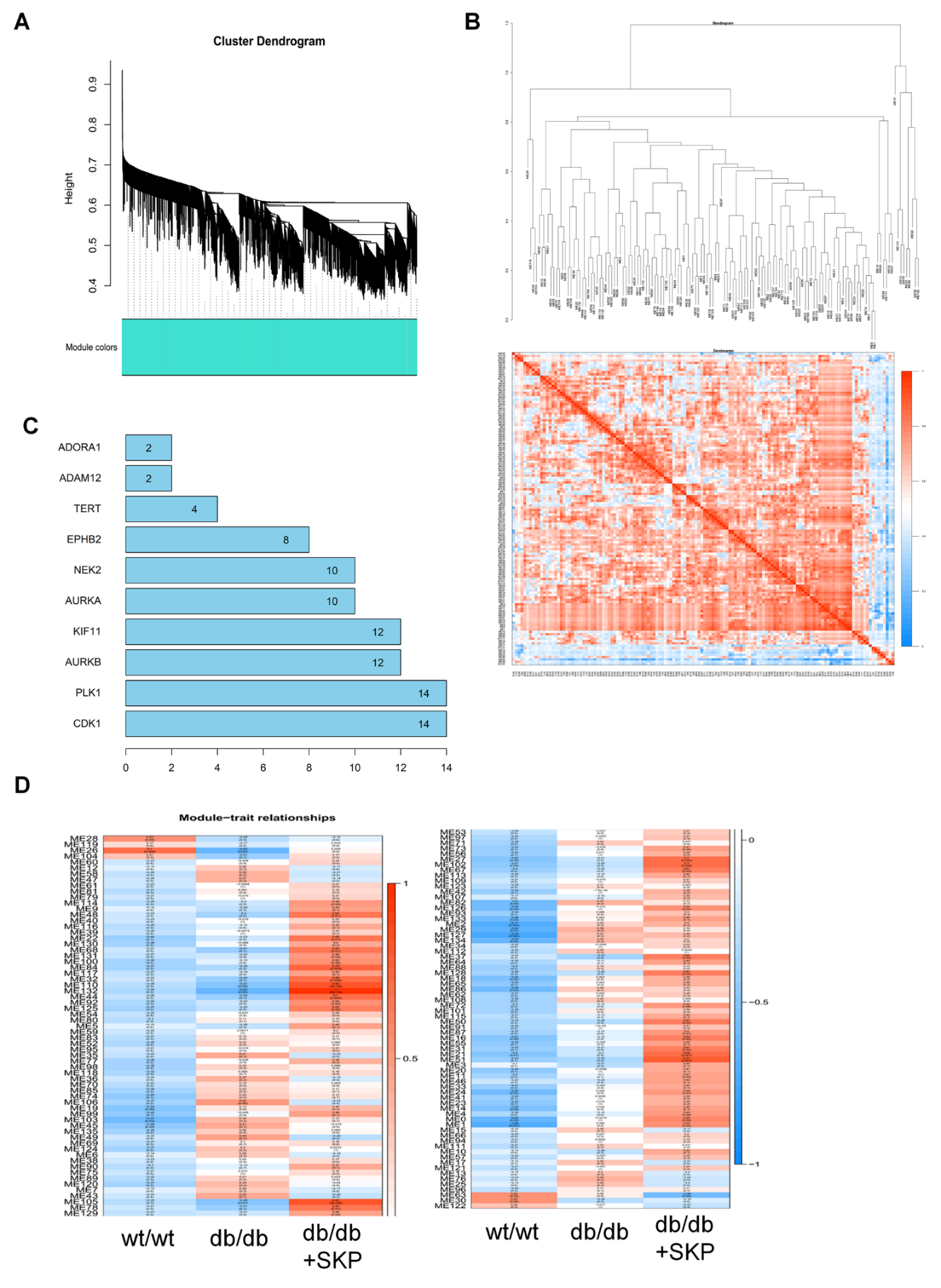


**Figure S1** (**A**) WGCNA module clustering performed with 3 groups of 12 samples and 20328 transcripts. (**B**) A heat map of correlation coefficients between gene modules (bottom) and clustering results (top). (**C**) visualization of PPI diagram based on Figure 5C. (**D**) A heat map of correlation coefficient between gene module and phenotype.


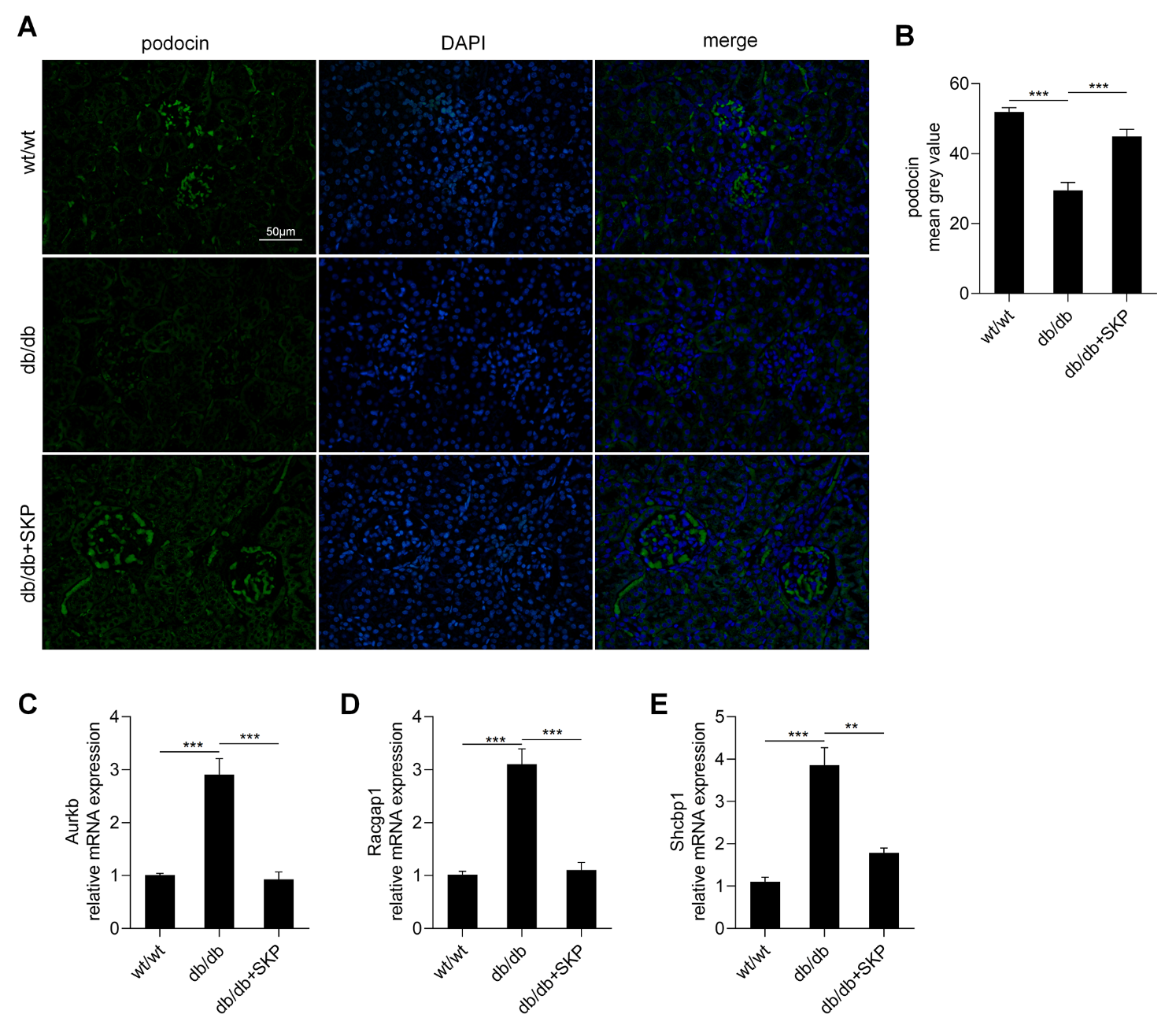


**Figure S2** (**A**) podocin expression in glomeruli by immunofluorescence staining. Original magnification × 400. (**B**) fluorescence intensity of podocin measured by image J. (**C-E**) RT-qPCR of AURKB (**C**), RacGAP1 (**D**) and shcbp1(**E**) in the kidney. All values are shown as mean ± SEM, with levels of significance determined by ANOVA and a subsequent Tukey test. ^**^*P* < 0.01 and ^***^*P* < 0.001 vs. the indicated groups.
